# ASK1 signalling regulates brown and beige adipocyte function

**DOI:** 10.1038/ncomms11158

**Published:** 2016-04-05

**Authors:** Kazuki Hattori, Isao Naguro, Kohki Okabe, Takashi Funatsu, Shotaro Furutani, Kohsuke Takeda, Hidenori Ichijo

**Affiliations:** 1 The laboratory of Cell Signaling, Graduate School of Pharmaceutical Sciences, The University of Tokyo, 7-3-1 Hongo, Bunkyo-ku, Tokyo 113-0033, Japan; 2 The laboratory of Bioanalytical Chemistry, Graduate School of Pharmaceutical Sciences, The University of Tokyo, 7-3-1 Hongo, Bunkyo-ku, Tokyo 113-0033, Japan; 3 JST, PRESTO, 4-1-8 Honcho, Kawaguchi, Saitama 332-0012, Japan; 4 Division of Cell Regulation, Graduate School of Biomedical Sciences, Nagasaki University, 1-14 Bunkyo-machi, Nagasaki 852-8521, Japan

## Abstract

Recent studies suggest that adult humans have active brown or beige adipocytes, the activation of which might be a therapeutic strategy for the treatment of diverse metabolic diseases. Here we show that the protein kinase ASK1 regulates brown and beige adipocytes function. In brown or white adipocytes, the PKA-ASK1-p38 axis is activated in response to cAMP signalling and contributes to the cell-autonomous induction of genes, including Ucp1. Global and fat-specific ASK1 deficiency leads to impaired metabolic responses, including thermogenesis and oxygen consumption, at the cell and whole-body levels, respectively. Our data thus indicate that the ASK1 signalling axis is a regulator of brown and beige adipocyte gene expression and function.

## Abstract (web summary)

The protein kinase ASK1 has been linked to cellular stress responses. Here the authors show that ASK1 also regulates gene expression and activity of beige and brown adipocytes, and demonstrate adipocyte ASK1 has a physiological role in regulating thermogenesis in mice.

## Graphical abstract

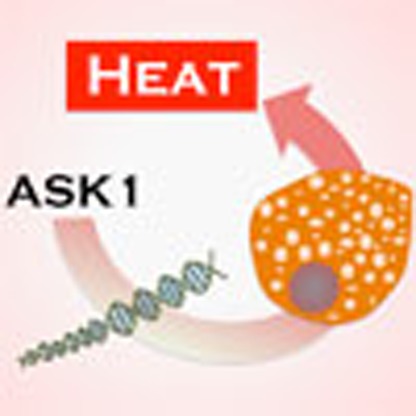

Brown adipose tissue (BAT) was thought to be one of the major contributors to energy homoeostasis in rodents, but it is only recently that the active form of BAT was proven to also exist in adult humans^1^,^2^,^3^,^4^. Although brown adipocytes, like white adipocytes, can store lipid droplets within the cell, brown adipocytes dissipate energy in the form of heat in response to several types of stimuli, including cold exposure^5^. Uncoupling protein 1 (Ucp1) is a key molecule that is specifically expressed in BAT and dissipates the mitochondrial electrochemical gradient to produce heat^6^,^7^. β-Adrenoceptors (βARs) are regarded as critical sensors to control lipid metabolism in adipocytes in response to catecholamine release from nerve endings. βARs consist of three subtypes: β_1_AR, β_2_AR and β_3_AR, but β_3_AR is selectively expressed in adipocytes^8^. βARs are members of the G protein-coupled receptor family. When coupled with the Gsα subunit, their activation leads to the activation of adenylyl cyclase to increase intracellular cAMP levels, which induces cAMP-dependent protein kinase (PKA) axis activation. A diverse array of thermogenic events is subsequently induced, including lipolysis, Ucp1 activation and Ucp1 induction^9^.

It has been thought that Ucp1 expression is restricted to BAT; however, recent studies have demonstrated that Ucp1-positive cells can be detected even in white adipose tissue (WAT) under certain circumstances. These cells are called ‘beige cells'^9^. It is well-known that β_3_AR agonist treatment, which can be considered as sympathetic hyperactivity, induces Ucp1 expression in subcutaneous WAT^10^,^11^. The emergence of Ucp1-positive beige cells in WAT is thought to have beneficial effects on metabolism^12^,^13^,^14^. Recent studies indicate that humans possess both classical brown adipocytes and beige adipocytes; hence, both cell types are potential therapeutic targets for metabolic diseases^15^,^16^,^17^,^18^,^19^.

Extensive studies have identified the critical transcriptional regulators involved in brown or beige adipocyte differentiation; however, the signalling molecules that precisely control the transcriptional system are largely unknown. The mitogen-activated protein kinase (MAPK) cascades are one of the major intracellular signalling pathways that are evolutionarily conserved in eukaryotic cells and regulate multiple cellular responses, including gene induction, cell death and lipid homoeostasis^20^. Ucp1 gene induction, especially in brown adipocytes, is partly regulated by the MKK6-p38α pathway 21,22,23. However, how the upstream kinase cascades regulate such pathways are poorly understood.

Apoptosis signal-regulating kinase 1 (ASK1) is one of the MAP3Ks in the JNK and p38 axes, and it responds to various stresses such as oxidative stress, ER stress and calcium influx^24^,^25^,^26^. It has been reported that ASK1 is expressed in human adipose tissue, but the functions and physiological relevance of ASK1 in adipose tissue remain largely unknown^27^,^28^. Furthermore, little is known about the function of MAPK cascades in adipose tissue and the relationship between MAPK cascades and cAMP signalling. We therefore attempted to elucidate the significance of the ASK1 cascade in adipose tissue.

Here, we show that PKA-ASK1-p38 cascade is involved in brown and beige adipocyte function, including thermogenesis, in part by regulating Ucp1 induction.

## Results

### The function of ASK1 in brown adipocytes

**Figure 1:**
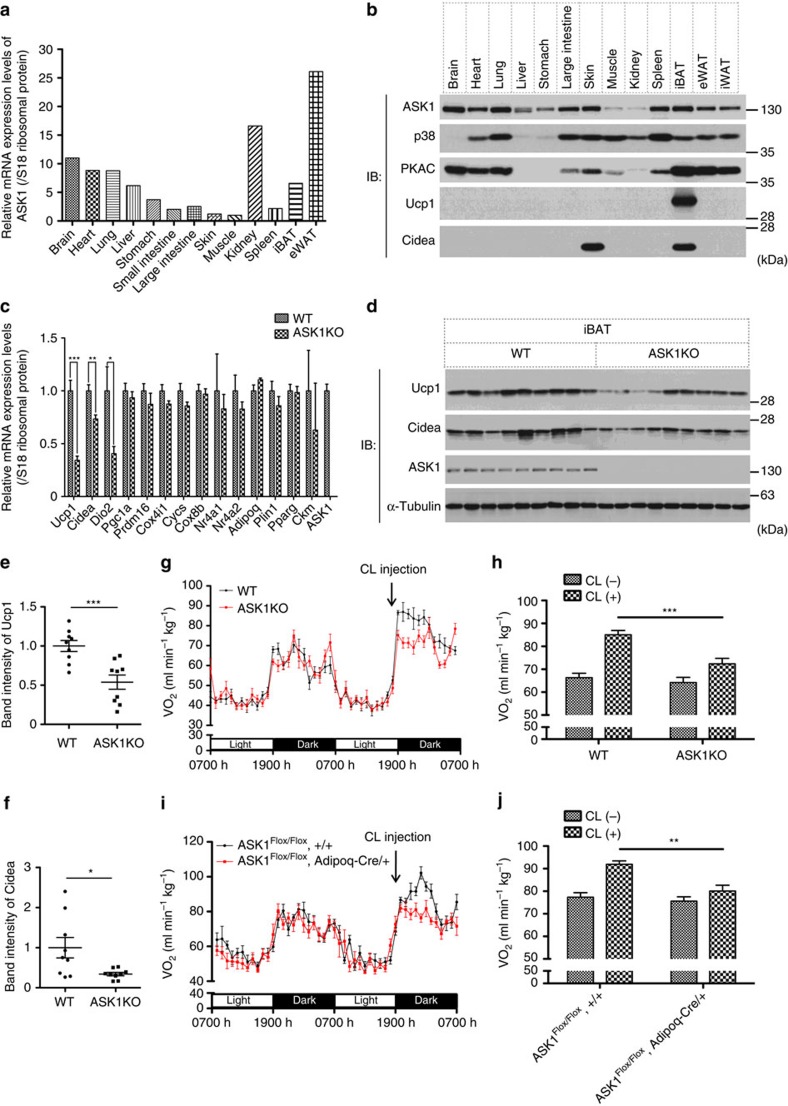
BAT function is impaired in ASK1-deficient mice. (**a**) qRT–PCR analysis of RNA isolated from tissues of adult WT mice. (**b**) Western blotting analysis of proteins isolated from tissues of adult WT mice. (**c**) qRT–PCR against indicated genes in iBAT (*n*=9). (**d**) Western blot against Ucp1 and Cidea in iBAT (*n*=9). (**e**,**f**) Band intensities of Ucp1 (**e**) and Cidea (**f**) were plotted (*n*=9). (**g**) VO_2_ of mice treated with CL316,243 (*n*=6). CL316,243 was injected i.p. at ∼1845 h. (**h**) Six-hour average of VO_2_ from 1900 h to 2400 h with or without CL316,243 injection (*n*=6). (**i**) VO_2_ of mice treated with CL316,243 (*n*=6, 8). CL316,243 was injected i.p. at ∼1845 h. (**j**) Six-hour average of VO_2_ from 1900 h to 2400 h with or without CL316,243 injection (*n*=6, 8). (**b**,**d**) The same amount of protein was loaded in each lane. (**c**,**e**,**f**) *P<0.05, **P<0.01, ***P<0.001 by unpaired two-tailed Student's *t*-test. (**h**,**j**) **P<0.01, ***P<0.001 by two-way ANOVA followed by Bonferroni's multiple comparisons test. All data are represented as the mean±s.e.m.

We first performed quantitative RT–PCR analysis to measure ASK1 mRNA abundance in each tissue. As previously reported^29^, ASK1 mRNA is ubiquitously expressed in all tissues examined including WATs and BATs (Fig. 1a), and the expression of ASK1 protein in adipose tissues has also been confirmed by immunoblotting (Fig. 1b).

To elucidate the potential function of ASK1 in adipose tissues, DNA microarray analysis was performed using interscapular BAT (iBAT) from wild-type (WT) and ASK1-deficient mice. We found that the expression levels of several brown adipocyte-selective genes including Ucp1 and Cidea were reduced in ASK1-deficient iBAT (Supplementary Table 1). Quantitative RT–PCR analyses were performed to confirm the results of the microarray analysis and revealed that expression levels of Ucp1, Cidea and Dio2, which are brown adipocyte-selective genes, were suppressed in ASK1-deficient iBAT (Fig. 1c). Expression levels of several other critical molecules for brown adipocyte differentiation, including Pparγ, Prdm16 and Pgc1α, were comparable between ASK1-deficient and WT iBAT (Fig. 1c). The protein expressions of Ucp1 and Cidea were also attenuated in ASK1-deficient iBAT (Fig. 1d), and the differences were statistically significant (Fig. 1e,f). These data suggest that ASK1 regulates expression of a repertoire of brown adipocyte-selective genes.

Because Ucp1 is critical for maintaining body temperature and metabolic homoeostasis, we tested the effects of ASK1 deficiency in a mouse model, specifically focusing on BAT function. We administered CL316,243, a β_3_AR-specific agonist, to mice because the increase in the CL316,243-dependent oxygen consumption rate (VO_2_) is known to derive from Ucp1 activity^30^. The VO_2_ of WT mice certainly increased after CL316,243 injection; however, ASK1-deficient mice exhibited only a modest increase compared with WT mice (Fig. 1g,h). The respiratory quotient was comparable after CL316,243 administration (Supplementary Fig. 1a,b). ASK1-deficient mice showed no significant aberrant phenotype under normal environmental conditions (Supplementary Fig. 1c–g); however, we found slight but significant differences in total cholesterol and free fatty acids in the sera of ASK1-deficient mice (Supplementary Fig. 1h,i). We also could not observe any clear alteration in the VO_2_ or respiratory quotient in a normal environment (Supplementary Fig. 1j,k). Lipolytic responses to CL316,243 were also comparable between the two genotypes (Supplementary Fig. 1l). These results suggest that ASK1-deficient BAT has defects in energy expenditure owing to reduced levels of Ucp1. The fact that ASK1-deficient mice showed vulnerability to cold shock in the fasted state also suggests the importance of ASK1 in maintaining metabolic homoeostasis (Supplementary Fig. 1m). Although there was no effect on adipose tissue mass under standard conditions, high-fat diet-induced increase of inguinal WAT (iWAT) and epididymal WAT (eWAT) ratio were enhanced in ASK1-deficient mice, suggesting the limited effect of ASK1 deficiency for adiposity (Supplementary Fig. 2a–e).

To examine the specific role of ASK1 in adipocytes, we generated adipocyte-specific ASK1-deficient mice, which showed absence of ASK1 specifically in BAT and WAT (Supplementary Fig. 3a–e). As is the case with whole-body ASK1-deficient mice, adipocyte-specific ASK1-deficient mice exhibited a suppressed VO_2_ increase in response to CL316,243 administration with reduced expression of Ucp1 in iBAT (Fig. 1i,j, Supplementary Fig. 3f,g). These findings are indicative of an adipocyte-specific role of ASK1 for brown adipocyte function.

**Figure 2:**
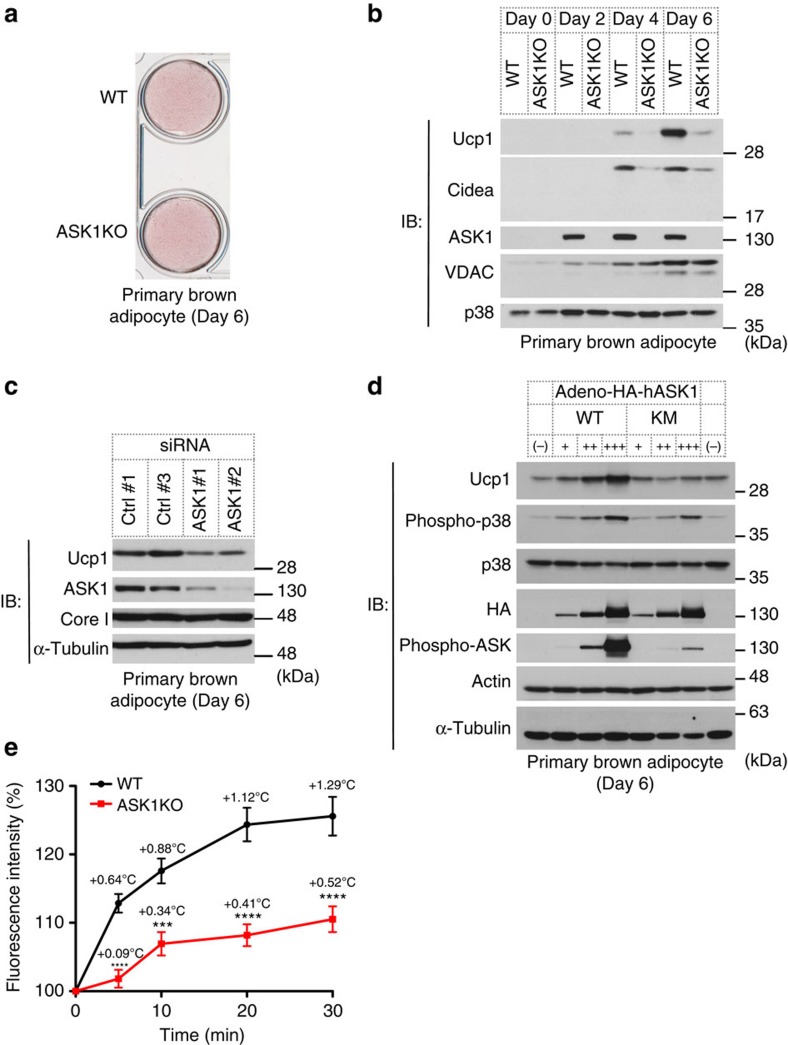
ASK1 is involved in brown adipocyte-selective gene expression. (**a**) Oil red O staining of differentiated brown adipocytes (day 6). (**b**) Western blot against indicated genes in brown adipocytes. The same amount of protein was loaded in each lane. (**c**) Western blot against indicated genes in brown adipocytes (day 6). (**d**) Western blot analysis to detect Ucp1 expression in differentiated adipocytes (day 6) in which WT or kinase-negative mutant (KM) ASK1 was overexpressed. (**e**) Intracellular thermometry using fluorescent nanogel thermometer (*n*=11 (WT), *n*=22 (ASK1KO)). Estimated temperature change for each point is shown as an insert. ***P<0.001, ****P<0.0001 compared with WT by two-way RM ANOVA followed by Bonferroni's multiple comparisons test. Data are represented as the mean±s.e.m.

We next examined whether ASK1 functions in brown adipocytes cell autonomously by using primary cultured cells isolated from newborn mice. To evaluate the protein expression levels of brown adipocyte-selective genes, cell lysates were prepared every other day during the differentiation process. Almost every cell accumulated lipids in the cytosol on day 6, suggesting that adipocytes were fully differentiated. The extent of triglyceride accumulation measured by Oil red O staining was comparable between WT and ASK1-deficient adipocytes on day 6 (Fig. 2a). Ucp1 and Cidea, both of which are brown adipocyte-selective genes, were induced as differentiation progressed in WT adipocytes (Fig. 2b). ASK1 was clearly induced during the course of differentiation even though it was undetectable in pre-adipocytes on day 0 (Fig. 2b). The protein amounts of Ucp1 and Cidea were clearly reduced in ASK1-deficient brown adipocytes on day 6, which was comparable to the result of iBAT (Fig. 2b). Similar results were obtained with mRNA levels (Supplementary Fig. 4). Furthermore, ASK1 requirement for Ucp1 induction was also confirmed by siRNA-mediated knockdown experiment (Fig. 2c). Our data suggest that ASK1 is required for inducing brown adipocyte-selective genes. We also examined the effect of increased ASK1 expression on adipocyte differentiation using an adenovirus-mediated overexpression system. Primary cultured brown adipocytes were infected with adenoviruses on day 2, and exogenously expressed WT ASK1 or kinase-negative mutant (KM) ASK1 were detected on day 6 (Fig. 2d). Enzymatic activities of ASK1 and p38, which were assessed by measuring phospho-ASK or phospho-p38 signals, increased in a dose-dependent manner in adipocytes overexpressing ASK1-WT, but not ASK1-KM, and Ucp1 expression was induced in concert with ASK1-p38 pathway activation (Fig. 2d). Taken together, ASK1 can trigger Ucp1 induction in a kinase activity-dependent manner.

Several studies suggested that Ucp1 is critical for increasing oxygen consumption after treatment with βAR agonists^31^. Although thermogenesis is also supposed to be induced by treatment with βAR agonists, intracellular heat production was barely measurable because of limitations of the experimental system. We therefore used intracellular thermometry using hydrophilic fluorescent nanogel thermometers in differentiated brown adipocytes^32^. As expected, CL316,243 treatment-dependent thermogenesis was abolished in ASK1-deficient adipocytes, suggesting that ablation of Ucp1 in ASK1-deficient cells leads to the suppression of cellular thermogenesis(Fig. 2e).

### PKA-ASK1-p38 axis induces Ucp1 expression

**Figure 3:**
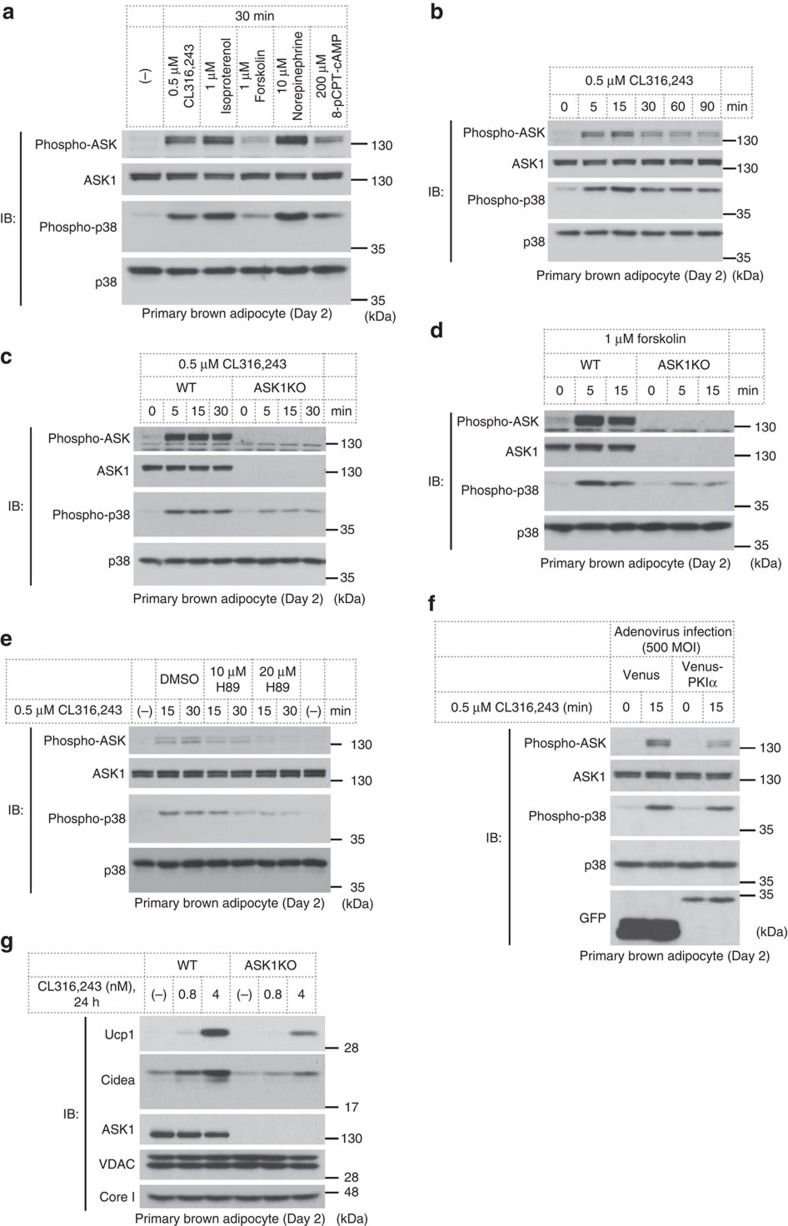
cAMP signalling activates the ASK1-p38 axis and leads to Ucp1 expression. (**a**) ASK1 and p38 activation in response to cAMP signalling in immature brown adipocytes. (**b**) Temporal change of ASK1 and p38 activation status. (**c**,**d**) Effect of ASK1 deficiency on cAMP signalling-dependent p38 activation. (**e**) Effect of H89 treatment on CL316,243-induced activation of the ASK1-p38 pathway. Cells were pretreated with H89 for 30 min before addition of CL316,243. (**f**) Activation levels of ASK1 in PKIα-infected cells. Cells were infected with adenovirus on day −2 and day 0 with 500 MOI. (**g**) Ucp1 and Cidea induction in immature brown adipocytes.

Previous studies have shown that p38 MAPK is activated by cAMP signalling in brown adipocytes, which is involved in Ucp1 expression^22^. We therefore examined the activation status of ASK1 using several compounds that increase intracellular cAMP levels in adipocytes. CL316,243 (a β_3_AR-specific agonist), isoproterenol (a pan-βAR agonist), forskolin (an adenylyl cyclase activator), norepinephrine (a pan-AR agonist) and 8-pCPT-cAMP (a cAMP analogue) all induced ASK1 and p38 activation in immature adipocytes (Fig. 3a). The activation lasted for at least 90 min, peaking at 15 min (Fig. 3b). The ASK1 response to cAMP signalling was also observed in 3T3-L1 adipocytes but not clearly in HEK293A cells (Supplementary Fig. 5a,b). Only a modest activation of p38 was observed in ASK1-deficient cells treated with CL316,243 or forskolin, indicating that cAMP signalling activates the ASK1-p38 axis in immature adipocytes (Fig. 3c,d). Phosphorylation levels of Ser133 in CREB and Ser660 in HSL, which are known to be regulated by PKA, were not reduced in ASK1-deficient cells, suggesting that PKA activity after treatment with CL316,243 or forskolin was not abrogated in ASK1-deficient cells^33^,^34^ (Supplementary Fig. 5c,d).

The importance of PKA function in cAMP signalling is well-known, and PKA is a potential upstream kinase of p38 in cAMP signalling in adipocytes^21^. Treatment with the well-known PKA inhibitor H89 blocked not only p38 but also ASK1 activation in response to CL316,243 (Fig. 3e). To exclude the non-specific action of H89, we used a protein kinase inhibitor (PKI) overexpression system^35^,^36^. Adenovirus-mediated overexpression of PKIα suppressed CL316,243-dependent ASK1 activation in immature adipocytes (Fig. 3f), indicating that CL316,243 activates ASK1 via cAMP-PKA signalling.

Previous studies have revealed that ASK1 is strongly activated by oxidative stress^26^. To examine the involvement of reactive oxygen species in cAMP-dependent ASK1 activation, we applied an antioxidant, *N*-acetyl-*L*-cysteine (NAC), before CL316,243 stimulation. H_2_O_2_-induced but not CL316,243-induced activation of ASK1 and p38 was abolished by treatment with NAC (Supplementary Fig. 5e), suggesting that PKA activates ASK1 in an oxidative stress-independent mechanism.

Furthermore, we evaluated the amounts of Ucp1 and Cidea protein in CL316,243-treated cells because cAMP signalling is regarded as a good inducer of their expression in brown adipocytes. Ucp1 and Cidea were clearly induced by CL316,243 treatment, but the induction level was reduced in ASK1-deficient cells (Fig. 3g). These data suggest that cAMP induces PKA-ASK1-p38 axis activation and leads to subsequent Ucp1 and Cidea expression. The decrease in the expressions of Ucp1 and Cidea in ASK1-deficient adipocytes was consistent with the results in ASK1-deficient iBAT (Fig. 1c,d).

**Figure 4:**
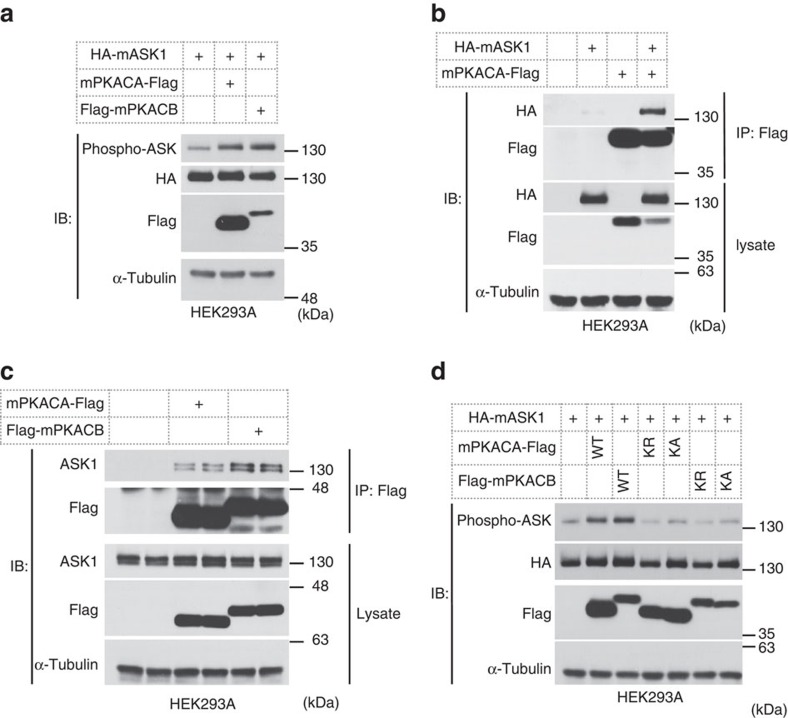
PKACs potentially interact with and activate ASK1. (**a**,**d**) Western blot analysis of ASK1 phosphorylation levels coexpressed with WT or kinase-inactive mutants (KR, KA) of PKA catalytic subunits. (**b**) Coimmunoprecipitation assay of exogenously expressed PKACA and ASK1. (**c**) Coimmunoprecipitation assay of exogenously expressed PKACA or PKACB and endogenous ASK1, performed in duplicate.

No other study has demonstrated the relevance of PKA and ASK1, while our observations suggest that PKA is an upstream kinase of ASK1. We attempted to analyse the interaction of these two molecules in HEK293A cells. ASK1 was cotransfected with PKACA and PKACB, the catalytic subunits of PKA, and the kinase activity of ASK1 was evaluated by measuring phospho-ASK1 signals. The results clearly showed that ASK1 was activated by both PKACs (Fig. 4a). We also detected the association between exogenously expressed PKACA and ASK1 in coimmunoprecipitation experiments (Fig. 4b). To confirm the interaction of PKACs and ASK1, we further analysed them in an immunoprecipitation experiment in which only PKACs were overexpressed. Consistent with the results shown in Fig. 4b, endogenous ASK1 was detected in the immune complex of PKACs (Fig. 4c). We next examined the role of kinase activity of PKACs in the activation of ASK1 observed in Fig. 4a. Kinase-inactive mutants of PKACs (Lysine72 to Arginine (KR) or Alanine (KA)) could not activate ASK1 (Fig. 4d). These results showed that PKACs can interact with and activate ASK1, supporting the conclusion that PKA is an upstream kinase of ASK1-p38 signalling in adipocytes (Fig. 3e,f).

### ASK1 is involved in browning

**Figure 5:**
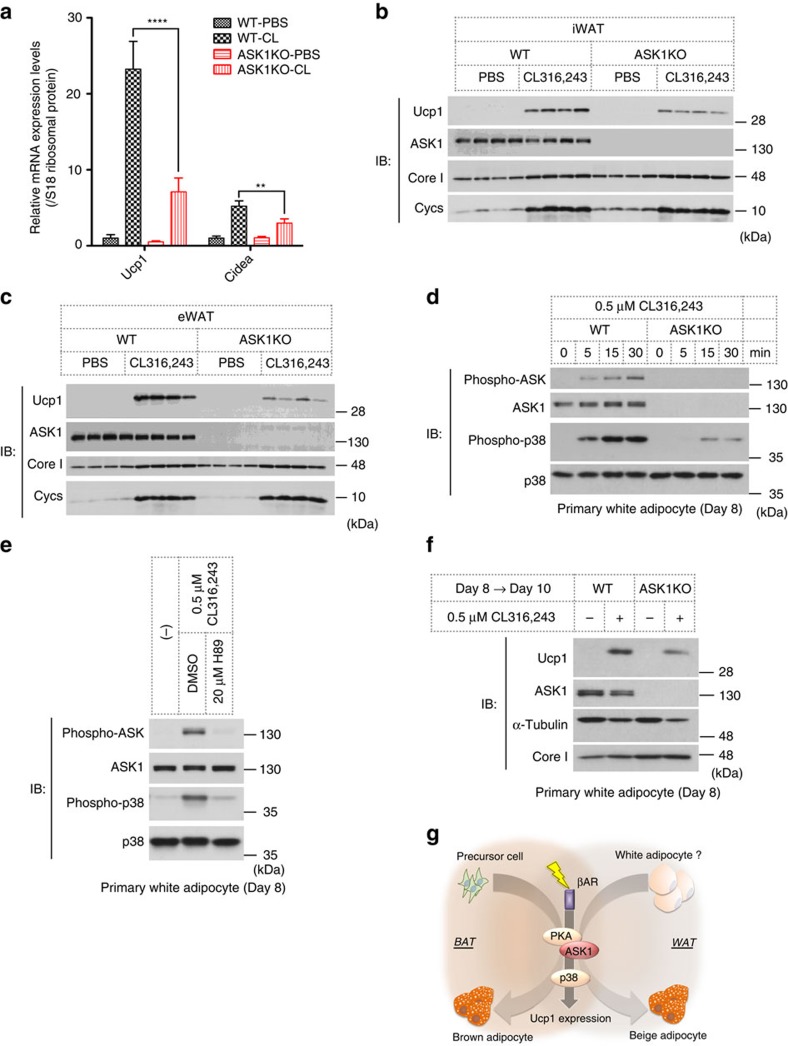
ASK1 is involved in browning. (**a**) CL316,243 or PBS was injected once a day for 2 days, and iWAT was subjected to qRT–PCR analysis (*n*=5). **P<0.01, ****P<0.0001 by two-way ANOVA followed by Bonferroni's multiple comparisons test. (**b**,**c**) CL316,243 or vehicle were injected once a day for 8 days, and iWAT (**b**) or eWAT (**c**) were subjected to western blot analysis (*n*=4). (**d**,**e**) Western blot analysis of phosphorylation of ASK1 and p38 in response to CL316,243 in differentiated white adipocytes with or without H89. (**f**) Western blot analysis of Ucp1 expression in response to CL316,243 in white adipocytes. (**g**) Proposed model for the role of ASK1 in BAT and WAT. Data are represented as the mean±s.e.m.

Our microarray analysis data indicated that the expression of brown adipocyte markers, such as Ucp1 and Cidea, was suppressed in ASK1-deficient eWAT, suggesting that ASK1 also regulates gene expression in beige adipocyte (Supplementary Table 2). To address the involvement of ASK1 in beige adipocyte development, we administered the β_3_AR-specific agonist CL316,243 to mice, because beige adipocytes are known to be induced by various stresses such as cold exposure or administration of βAR agonists^37^,^38^. CL316,243 administration markedly increased the amounts of Ucp1 and Cidea mRNA, but the inductions were significantly attenuated in ASK1-deficient mice (Fig. 5a). ASK1 contribution for Ucp1 induction was also confirmed in retroperitoneal WAT (rWAT) (Supplementary Fig. 6a). As an alternative strategy, we compared their protein expressions in iWAT and eWAT. The results clearly showed the reduced expression of Ucp1 in ASK1-deficient WATs, indicating that ASK1 positively regulates beige adipocyte development by inducing Ucp1 (Fig. 5b,c). Pgc1α, one of the critical molecules for browning^39^, was also suppressed in ASK1-deficient iWAT (Supplementary Fig. 6b), suggesting that the reduced Ucp1 expression was due at least in part to a decreased amount of Pgc1α. The mitochondrial markers Cox8b, Cox4i and Cox7a1 were all indeed induced by CL316,243 administration; however, their expression levels were unaltered in ASK-deficient tissues (Supplementary Fig. 6b). These data suggest that ASK1 induces a fraction of beige adipocyte-selective genes including Ucp1 in the same manner as it does in BAT.

We next sought to determine whether ASK1-dependent Ucp1 induction is a cell-autonomous phenomenon in white adipocytes. ASK1 was detectable in primary cultures of white adipocytes on day 8, at which almost all the cells accumulated lipid droplets within the cell (Supplementary Fig. 6c,d). The extent of lipid droplet formation within WT cells was equivalent to that in ASK1-deficient cells assessed by Oil red O staining (Supplementary Fig. 6d). CL316,243 clearly activated the ASK1-p38 pathway in mature white adipocytes in a similar fashion to brown adipocytes (Fig. 5d). Furthermore, these activations were abolished by pretreatment with the PKA inhibitor H89, indicating that the PKA-ASK1-p38 axis is also conserved in white adipocytes (Fig. 5e). Although Ucp1 protein expression was not observed in mature white adipocytes (day 8), we observed a marked increase in Ucp1 protein expression after treatment with CL316,243, and this induction was partially suppressed in white adipocytes derived from ASK1-deficient mice (Fig. 5f). These results suggest that ASK1 signalling also has a critical function in increasing the amount of Ucp1 in white adipocytes.

## Discussion

The data presented here show that the PKA-ASK1-p38 axis is activated in immature brown adipocytes and contributes to brown adipocyte-selective gene expression including Ucp1, which is essential for brown adipocyte function. We also demonstrated using genetically modified animals that ASK1 contributes to enhancing energy expenditure in brown adipocytes. On the basis of the gene expression profiles of ASK1-deficient iBAT or brown adipocytes, ASK1 seems to be involved in the expression of a specific subset, but not a broad spectrum, of genes (Fig. 1c, Supplementary Fig. 4). It is well-established that brown adipocytes and myoblasts arise from the same type of progenitors^40^. Given the whole-gene expression profile of ASK1-deficient iBAT (Fig. 1c), it is unlikely that ASK1 deficiency leads cells to differentiate into a cell type completely different from adipocytes. ASK1-deficient cells or mice, however, showed significant defects in heat production and oxygen consumption when treated with a β_3_AR agonist, presumably because Ucp1 is critical for dissipating energy in brown adipocytes (Figs 1g,h and 2e). The results obtained by using adipocyte-specific ASK1-deficient mice and primary culture of brown adipocytes strongly suggest that adipocyte-specific role of ASK1 contributes to the brown adipocyte function and the metabolic response in mice (Fig. 1i,j, Supplementary Fig. 3f,g).

We did not provide data revealing the downstream mechanism by which the PKA-ASK1-p38 axis induces gene expression. Pgc1α and/or ATF2 have been shown to be phosphorylated and to trigger Ucp1 expression in adipocytes on p38 activation^21^,^22^,^23^,^41^. We anticipate that Pgc1α and/or ATF2 might thus be involved in gene transcription downstream of the PKA-ASK1-p38 pathway.

Ucp1 expression was reduced in primary cultures of ASK1-deficient brown adipocytes even without any βAR agonist stimulation (Fig. 2b). This reduction is caused in part by the presence of 3-isobutyl-1-methylxanthine (IBMX), which increases intracellular cAMP levels, in the differentiation medium. Therefore, the PKA-ASK1-p38-Ucp1 axis may be activated to some extent during the differentiation process.

To date, the relationship between PKA and MAP3K remains largely unknown. One study showed that a member of the MAP3K family, TAK1, is involved in osteoclast differentiation through phosphorylation by PKA^42^. We argued here that many types of PKA activators could activate ASK1 (Fig. 3a), and ASK1 kinase activity is regulated by PKA in a kinase activity-dependent manner (Fig. 4d). Although the interaction of these two kinases was clearly detected (Fig. 4b,c), the detailed mechanism remains to be determined. The fact that NAC did not inhibit cAMP signalling-dependent ASK1 activation suggests that the activation mechanism is independent of reactive oxygen species (Supplementary Fig. 5e), which has been extensively examined in ASK1 signalling so far^43^. Our data suggest that cAMP signalling-dependent ASK1 activation is not clearly observed in other types of cells except for adipocytes (Supplementary Fig. 5b). The cell-type-specific mechanisms and functions of the PKA-ASK1 axis are important matters to be elucidated.

In addition to the analysis using brown adipocytes, we showed that PKA-ASK1-p38 signalling is conserved in mature white adipocytes. Treatment with β_3_AR agonist induces Ucp1 protein expression, which is one aspect of ‘browning', in mature white adipocytes. A recent topic of debate is whether beige adipocytes are derived from precursor cells or differentiated white adipocytes^44^,^45^. In our experiments, almost all cells had differentiated into mature white adipocytes on day 8, as evidenced by the presence of lipid droplets (Supplementary Fig. 6d), suggesting that only a few remaining precursor cells exist. Thus we suppose that Ucp1 is induced within mature white adipocytes in an ASK1 pathway-dependent manner.

As we mentioned above, ASK1 induction of brown adipocyte-selective gene expression was restricted to a specific set of genes. Similarly, inductions of only limited subsets of genes were affected in ASK1-deficient WAT in the βAR agonist-induced browning model (Supplementary Fig. 6b). Although the range of ASK1-induced gene expression is limited in both brown and beige adipocytes, there is no doubt that ASK1 is required for Ucp1 induction, which is essential for the physiological function of brown and beige adipocytes. The strategy of increasing the number of brown or beige adipocytes to combat metabolic diseases has recently been studied intensively. We believe that our results offer novel insight into brown or beige adipocyte function, which might be useful for developing therapeutic strategies aimed at enhancing brown/beige adipocyte thermogenic activity.

## Methods

### Antibodies and reagents

A polyclonal antibody to phospho-ASK1 (Thr838) was established as described previously^46^ and has been extensively validated. The antibody to Flag-tag (1E6, 1:1,000 or 1:10,000) and HA-tag (3F10, 1:1,000 or 1:10,000) were purchased from Wako and Roche, respectively. Phospho-specific antibodies to p38 (Thr180/Tyr182) (#9211, 1:5,000), HSL (Ser660) (#4126, 1:20,000) and CREB (Ser133) (#9191, 1:5,000) were purchased from Cell Signalling. Antibodies to p38 (#9228, 1:5,000), HSL (#4107, 1:2,000) and CREB (#9104, 1:5,000) were also purchased from Cell Signalling. Antibodies to Ucp1 (ab10983, 1:2,000 or 1:10,000), VDAC (ab14734, 1:5,000), PKAC (ab26322, 1:10,000) and ASK1 (ab45178, 1:10,000) were purchased from Abcam. Antibodies to Cidea (sc-8730-R, 1:2,000), α-tubulin (YL1/2, 1:20,000) and p38 (C20G, 1:2,000, used only in the experiments depicted in Supplementary Fig. 5a) were purchased from Santa Cruz Biotechnology; anti-Core I antibody (459140, 1:20,000) was purchased from Invitrogen; anti-Cytochrome C (556433, 1:10,000) was purchased from BD Pharmingen; and anti-actin antibody (A3853, 1:10,000) was purchased from Sigma.

CL316,243 (sc-203895) was purchased from Santa Cruz Biotechnology. Forskolin (F6886), isoproterenol (I6504), norepinephrine (A9512) and Oil red O (O0625) were purchased from Sigma. H89 (#9844S) was purchased from Cell Signalling. 8-pCPT-cAMP (039-18121) and H_2_O_2_ (081-04215) were purchased from Wako. NAC was purchased from Sigma (A8199) or Wako (017-05131).

### Cell culture and immunoblotting analysis

HEK293A cells were purchased from Invitrogen and were cultured in DMEM high-glucose containing 10% fetal bovine serum (FBS) in a 5% CO_2_ atmosphere at 37 °C.

3T3-L1 cells, a kind gift from Dr Shin-Ichiro Takahashi (The University of Tokyo), were cultured in DMEM high-glucose containing 10% calf serum and penicillin G. For differentiation, cells were plated and grown to 100% confluence (day −2). Cells were exposed to differentiation induction medium (DMEM high-glucose containing 10 μg ml^−1^ insulin, 250 nM dexamethasone, 0.5 mM IBMX, 100 U ml^−1^ penicillin G, 10% FBS) on day 0. The medium was changed to differentiation enhancement medium (DMEM high-glucose containing 10 μg ml^−1^ insulin, 100 units ml^−1^ penicillin G, 10% FBS) on day 2, day 4 and day 6.

Cells were tested for the presence of mycoplasma.

Cell extracts and immunoprecipitates were resolved by SDS-PAGE and electroblotted onto polyvinylidene difluoride membranes. The membranes were blocked with 2% skim milk in TBS-T (50 mM Tris-HCl, 150 mM NaCl and 0.05% Tween20, pH 8.0) and then probed with appropriate antibodies. Antibody-antigen complexes were detected using the ECL system. Signal intensities were measured by an image densitometer (ImageJ).

Adenoviruses encoding HA-tagged ASK1-WT and -KM mutant were constructed as described previously^47^. PKIα gene was cloned into pAd/CMV/V5 as described by the manufacturer (Invitrogen).

siRNA-mediated knockdown of ASK1 was performed at day 0 using Lipofectamine RNAiMAX Reagent (Life Technologies). siRNAs were purchased from Life Technologies and the sequences of the siRNAs used in this study are as follows: mouse ASK1 #1, 5′-AAUUGCAGUCUGCACAGCCUUUCGG-3′; mouse ASK1 #2, 5′-AAAUGCGUAAUGAAACUUCACGUGG-3′. Stealth RNAi siRNA Negative Control Med GC #1 and #3 were used as controls.

Representative data are shown in all immunoblotting analyses and more than two additional experimental replicates showed similar results.

### Primary cell culture

Primary brown fat stromal vascular fraction (SVF) from newborn male/female C57BL/6 J mice was obtained by the following procedure. Dissected iBAT was washed, minced and digested with collagenase (Wako) for 30 min at 37 °C. Digested tissue was filtered through a 100 μm cell strainer, and then the flow-through was centrifuged at 1,000 r.p.m. for 5 min. The pellet was washed once and resuspended in culture medium (DMEM high-glucose containing 20% FBS).

For differentiation, SVF cells were plated and grown to 100% confluence (day −2). Cells were exposed to differentiation induction medium (DMEM high-glucose containing 1 nM triiodo-L-thyronine (T3), 20 nM insulin, 5 μM dexamethasone, 0.125 mM indomethacin, 0.5 mM IBMX, 1 μM rosiglitazone, 20 mM HEPES-NaOH (pH 7.4), 20% FBS) on day 0. The medium was changed to differentiation enhancement medium (DMEM high-glucose containing 1 nM T3, 20 nM insulin, 20 mM HEPES-NaOH (pH 7.4), 20% FBS) on days 2 and 4.

Primary white fat SVF from 8- to 12-week-old male C57BL/6 J mice was obtained by the following procedure. Dissected inguinal WAT was washed, minced and digested with collagenase (Wako) and dispase II (Wako) for 1 h at 37 °C. Digested tissue was filtered through a 100 μm cell strainer, and then the flow-through was centrifuged at 1,000 r.p.m. for 10 min. The pellet was washed once and resuspended in culture medium (DMEM/Nutrient mixture F-12 HAM containing 10% FBS).

For differentiation, SVF cells were plated and grown to ∼100% confluence. Cells were exposed to differentiation induction medium (DMEM/F-12 HAM containing 1 nM T3, 5 μg ml^−1^ insulin, 5 μM dexamethasone, 0.125 mM indomethacin, 0.5 mM IBMX, 10% FBS) on day 0. The medium was changed to differentiation enhancement medium (DMEM/F-12 HAM containing 1 nM T3, 5 μg ml^−1^ insulin, 10% FBS) on day 2 and day 5.

### Oil red O staining

Mature adipocytes were washed with PBS and fixed in 10% formalin for 10 min. Cells were washed with PBS and rinsed with 60% isopropanol followed by staining with freshly prepared Oil red O solution. After rinsing with 60% isopropanol and PBS, the image was scanned.

### Quantitative PCR analysis

Total RNA was isolated from tissues or cells using Isogen (Wako) and reverse transcribed with ReverTra Ace qPCR RT Master Mix with gDNA Remover (Toyobo). Primers were designed using the Universal Probe Library Assay Design Center (Roche). Quantitative reverse transcription-PCR was carried out using a LightCycler 96 (Roche) or Prism 7000 (ABI) using SYBR Green PCR Master Mix. Data were normalized to S18. cDNAs purchased from GenoStaff were used to measure ASK1 expression levels in the different tissues shown in Fig. 1, and expression levels in muscle were assigned to a value of 1. Primer sequences are listed in Supplementary Table 3.

### DNA microarray

Micorarray hybridization and scanning were performed by Miltenyi Biotec using Agilent Whole Mouse Genome Oligo Microarrays 8 × 60K. Each sample was a mixture of total RNA derived from three 10-week-old male mice. The data set generated in this study is available online at NCBI GEO (GSE76660).

### Animals

C57BL/6 J strain 8- to 10-week-old male mice, bred in our facility, were used in all experiments unless otherwise indicated. The ASK1-deficient mice have been described previously^48^. Serum FFA, TAG and total cholesterol were measured in SRL, and blood glucose levels were monitored using MEDISAFE MINI (Terumo). CL316,243 was administrated at 0.1 mg kg^−1^ weight to detect Ucp1 in WAT (subcutaneously) or to measure serum FFA (intraperitoneally (i.p.)), and at 1 mg kg^−1^ weight to measure VO_2_ (i.p.). Animals were randomly chosen for each experiment.

A 12-kb fragment containing exon 15 and exon 16 was used for construction of the targeting vector. The loxP and FRT-flanked Neo cassette was inserted downstream of exon 15, and single loxP site was inserted upstream of exon 15. The targeting vector was injected into the C57BL/6-derived ES cell line, RENKA, resulting in the generation of ASK1^Neo/+^ mice. The neomycin cassette was excised by breeding it with B6-Tg(CAG-FLPe)36 mice and ASK1^Flox/+^ mice were generated^49^. ASK1^Flox/Flox^ mice were produced by breeding ASK1^Flox/+^ mice. B6;FVB-Tg(Adipoq-cre)1Evdr/J mice were purchased from the Jackson Laboratory.

Animal experiments were performed according to procedures approved by the Graduate School of Pharmaceutical Sciences, The University of Tokyo.

WT mice and ASK1-deficient mice have been bred separately; however, littermate control mice have been used to compare with adipocyte-specific ASK1-deficient mice.

### Indirect calorimetry

Whole-body O_2_ consumption was measured using an open-circuit four-chamber indirect calorimetry system (Muromachi). All the 8- to 10-week-old male mice had *ad libitum* access to food and water, and data were recorded for a 24-h period with light from 0700 h to 1900 h.

### High-fat diet-induced obesity model

WT and ASK1-deficient male mice were fed normal diet or high-fat diet (CLEA Japan) for 10 weeks from the age of 16 weeks and body weight was monitored every week. Mice were killed and each tissue was weighed. Animals were randomly chosen for each experimental group.

### Intracellular thermometry

Fluorescent nanogel thermometer was introduced into the cytosol of primary brown adipocytes (day 6) by a microinjection technique, and fluorescence intensity of every single cell was obtained before and after CL316,243 (at a final concentration of 0.5 μM) administration using fluorescence microscopy^32^. Data were normalized to the fluorescence intensity of time 0.

### Statistics

All data are expressed as the mean±s.e.m. Unpaired two-tailed Student's *t*-test, two-way ANOVA or two-way RM ANOVA followed by Bonferroni's multiple comparisons test were used. Statistical analyses were performed using GraphPad Prism.

No statistical methods were used to determine sample size in advance. No samples or animals were excluded from the analysis in most of the experiments; however, the data sets in which the average of β_3_ agonist-induced VO_2_ increase of control mice were less than 10 ml min^−1^ kg^−1^ were excluded in indirect calorimetry experiments, which was pre-established. The investigators were not blinded to the group allocation during the experiment.

## Additional information

**How to cite this article**: Hattori, K. *et al.* ASK1 signalling regulates brown and beige adipocyte function. *Nat. Commun.* 7:11158 doi: 10.1038/ncomms11158 (2016).

## Supplementary Material

Supplementary Information Supplementary Figures 1-7 and Supplementary Tables 1-3
