# Supplementary material for: ASK1 signalling regulates brown and beige adipocyte function
Source: Nat Commun. 2016 Apr 5;7:11158. doi: 10.1038/ncomms11158 (PMC4822029; doi:10.1038/ncomms11158)
Supplement: Supplementary Information — Supplementary Figures 1-7 and Supplementary Tables 1-3 [file ncomms11158-s1.pdf]

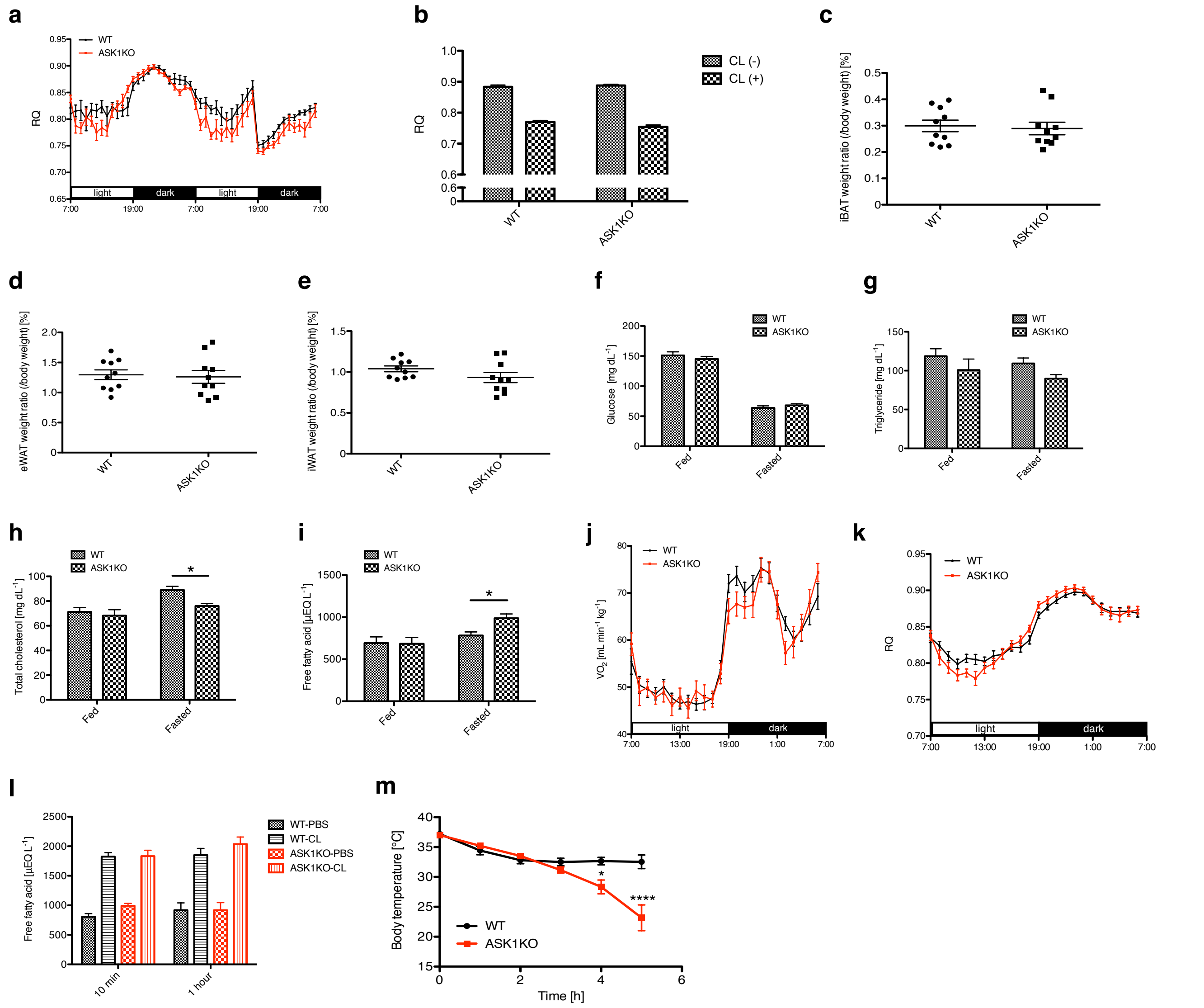

Supplementary Fig. 1 Phenotype of ASK1-deficient mice

(a, b) RQ of mice treated with CL316,243 (N=6) (a). CL316,243 was injected intraperitoneally at approximately 18:45. Six-hour average of RQ from 19:00 to 24:00 with or without CL316,243 injection (N=6) (b).

(c, d, e) Ratio of iBAT (c), eWAT (d), and iWAT (e) weight to body weight (N=10).

(f, g, h, i) Glucose (f), triglyceride (TAG) (g), total cholesterol (h), free fatty acid (FFA) (i) in serum of 10-12-week-old male mice under fed or 16-hour fasted conditions. N=19 (WT, Fed), 16 (WT, Fasted), 18 (ASK1KO, Fed), 17 (ASK1KO, Fasted) (f). N=14 (WT, Fed), 16 (WT, Fasted), 13 (ASK1KO, Fed), 17 (ASK1KO, Fasted) (g, h, i). *P<0.05 by two-way ANOVA followed by Bonferroni multiple comparisons test.

(j, k) Oxygen consumption rate (VO_2) (j) and respiratory quotient (RQ) (k) (N=8). Data are three-day averages for each mouse.

(l) Serum free fatty acid levels 15 minutes or 1 hour after injection of CL316,243 (N=5 for PBS, N=8 for CL). Mice were fasted for 16 hours before injection.

(m) Core body temperature in wild-type (N=5) and ASK1-deficient mice (N=8). Time point 0 was after food and water deprivation for 16 hours, and the start point of cold exposure. *P<0.05, ****P<0.0001 compared with wild-type by two-way RM ANOVA followed by Bonferroni multiple comparison test.

Data are represented as the mean ± s.e.m.

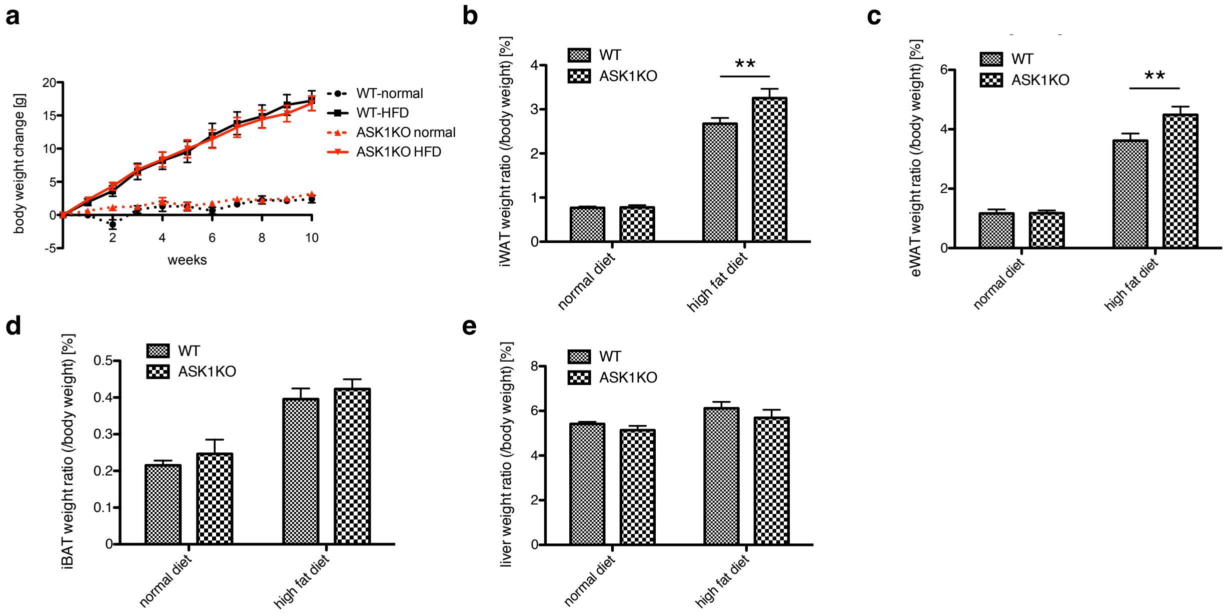

Supplementary Fig. 2 High-fat diet-induced obesity model

(a) Body weight change of wild-type and ASK1-deficient mice fed either normal or high-fat diet (HFD) starting at 16 weeks of age (N=8).

(b, c, d, e) Ratio of iWAT (b), eWAT (c), iBAT (d), liver (e) weight to body weight fed normal or high-fat diet for 10 weeks (N=8).

**$P<0.01$ by two-way ANOVA followed by Bonferroni multiple comparisons test.

Data are represented as the mean ± s.e.m.

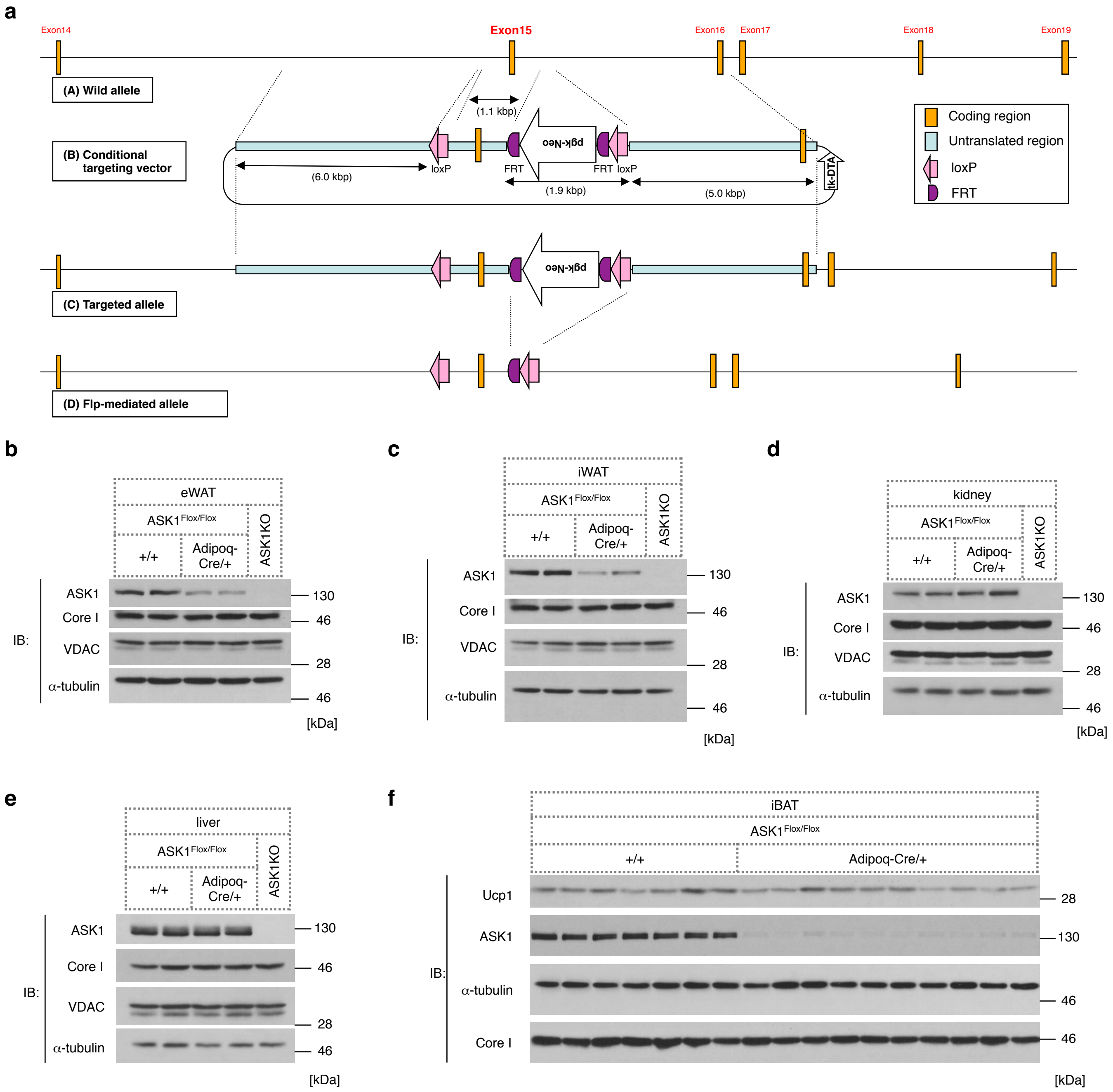

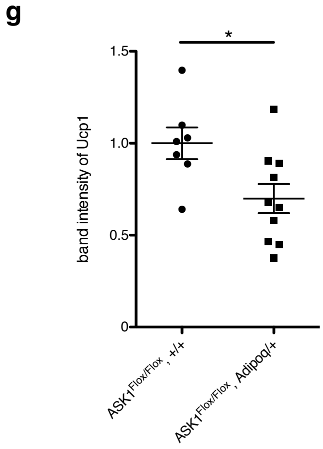

Supplementary Fig. 3 Adipocyte-specific ASK1-deficient mice
(a) Schematic model of ASK1$^{Flox/Flox}$ mice generation.
(b, c, d, e) ASK1 protein expression in eWAT (b), iWAT (c), kidney (d) and liver (e) of 9-week-old male mice.
(f) Western blot against Ucp1 in iBAT (N=7, 10).
(g) Band intensities of Ucp1were plotted (N=7, 10).
(b, c, d, e, f) The same amount of protein was loaded in each lane.

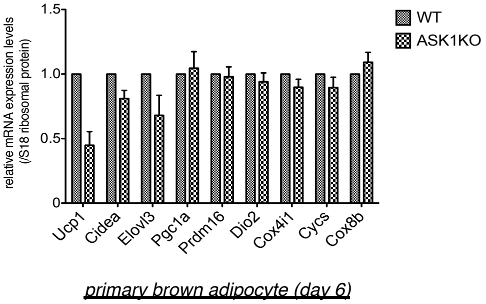

Supplementary Fig. 4 Gene expression profile of brown adipocytes derived from ASK1-deficient mice
qRT-PCR of indicated genes in differentiated brown adipocytes (day 6). Expression levels in ASK1-deficient adipocytes were normalized to that in wild-type adipocytes in each experiment (N=5).
Data are represented as the mean ± s.e.m.

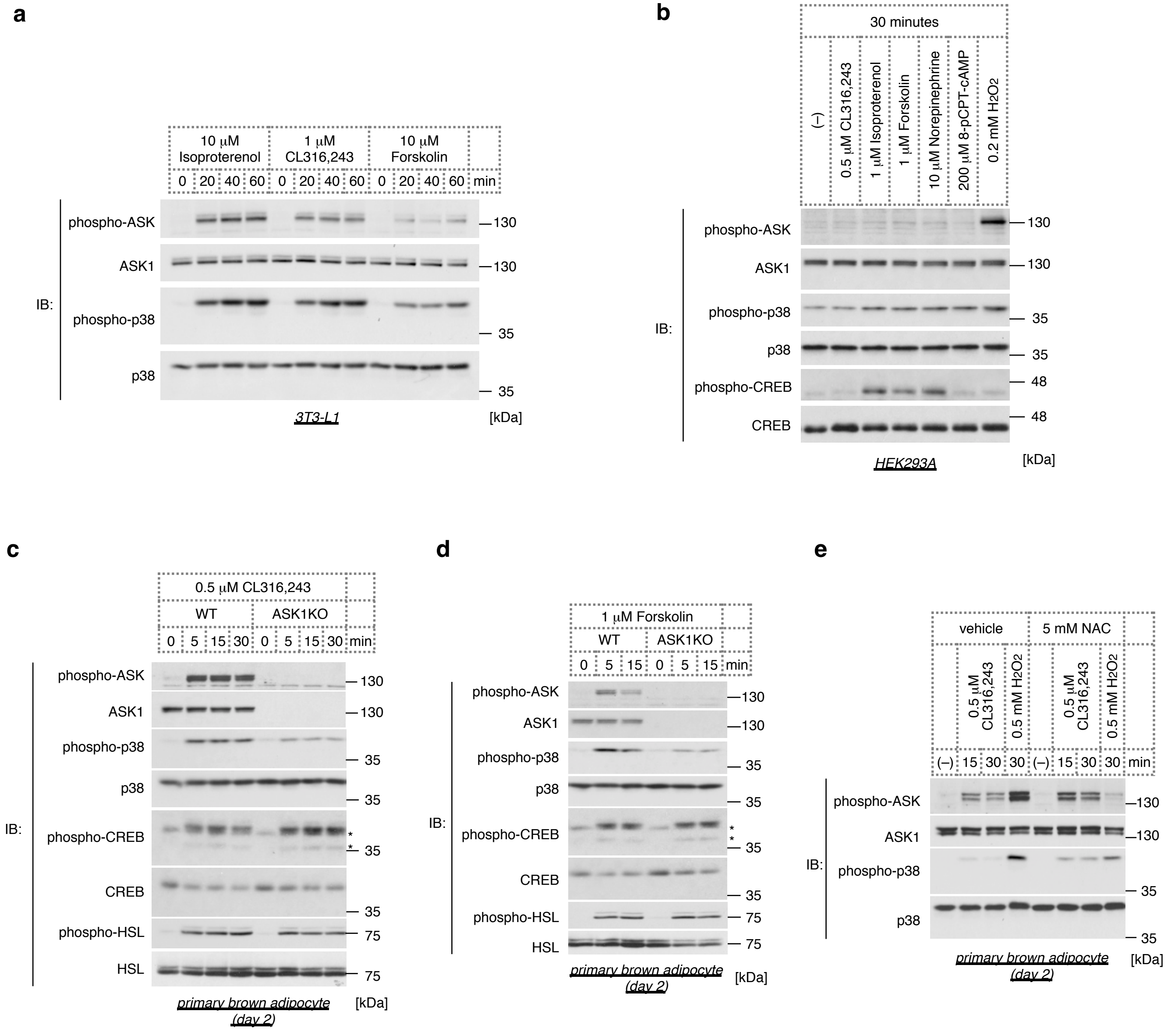

Supplementary Fig. 5 cAMP signaling responses in several cell types

(a) ASK1 and p38 activation in response to cAMP signaling in mature 3T3-L1 adipocytes (a) or in HEK293A cells (b).

(c, d) Effect of ASK1 deficiency for phospho-CREB or phospho-HSL levels.

(e) ASK1 and p38 activation levels in immature brown adipocytes pretreated with antioxidant NAC for 30 minutes before CL316,243 or H_2O_2 was applied.

Asterisks indicate nonspecific bands.

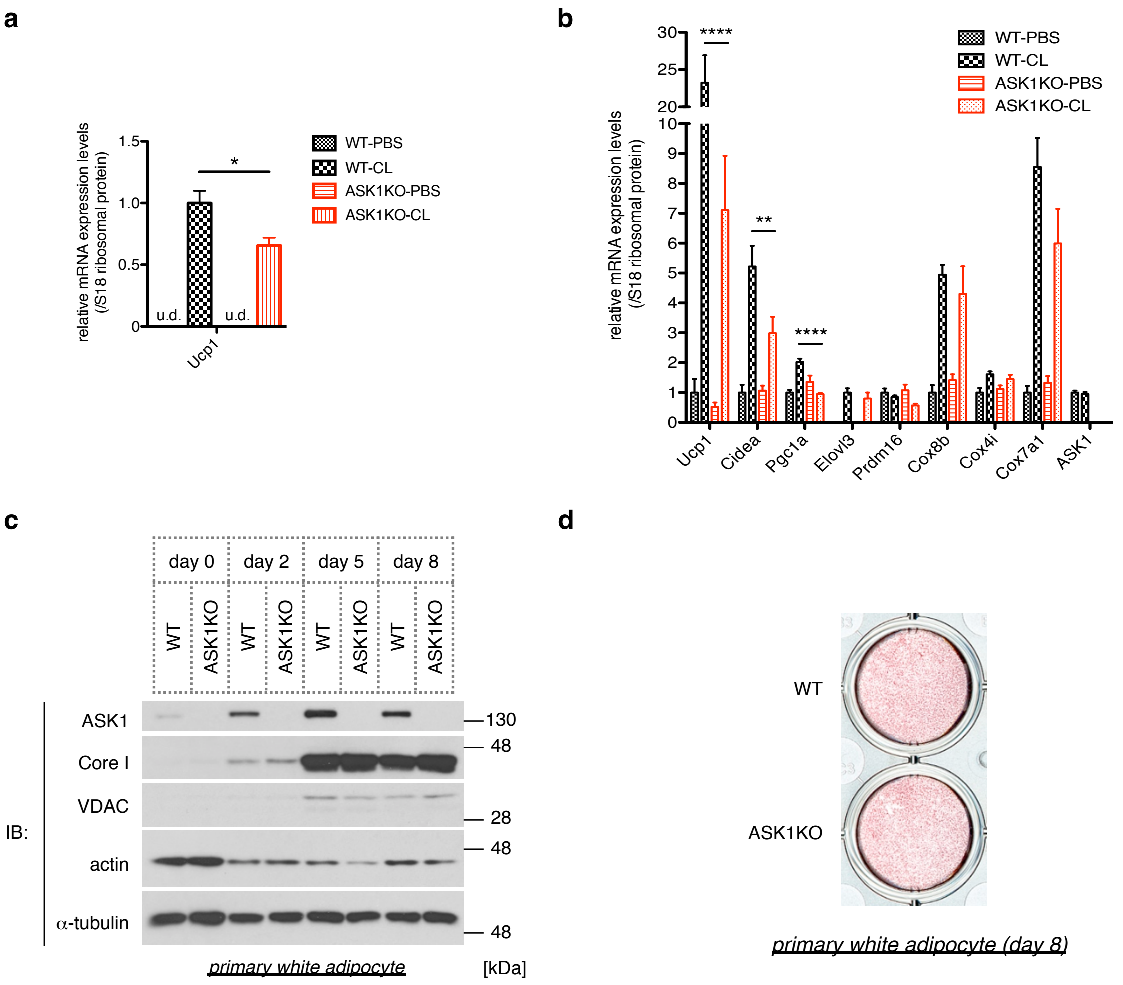

Supplementary Fig. 6 Characterization of ASK1-deficient white adipose tissue and white adipocyte

(a) Either CL316,243 or vehicle was injected once a day for 2 days, and rWAT of wild-type and ASK1-deficient mice were subjected to qRT-PCR analysis (N=4). *P<0.05 by unpaired two-tailed t-test. u.d.: undetectable.

(b) Either CL316,243 or vehicle was injected once a day for 2 days, and iWAT of wild-type and ASK1-deficient mice were subjected to qRT-PCR analysis (N=5). **P<0.01, ****P<0.0001 by two-way ANOVA followed by Bonferroni multiple comparisons test.

(c) Western blot analysis in white adipocytes. The same amount of protein was loaded in each lane.

(d) Oil red O staining of differentiated white adipocytes (day 8).

Data are represented as the mean ± s.e.m.

Fig. 1b

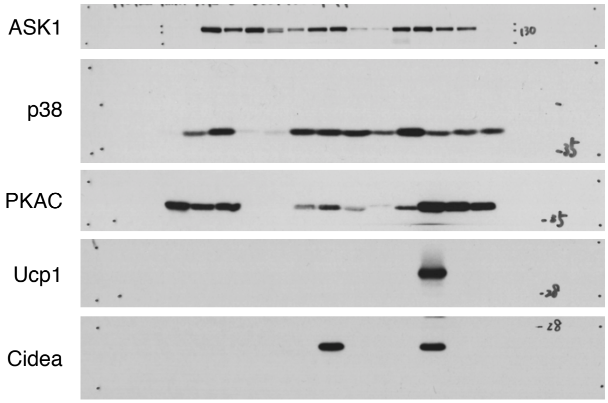

Fig. 1d

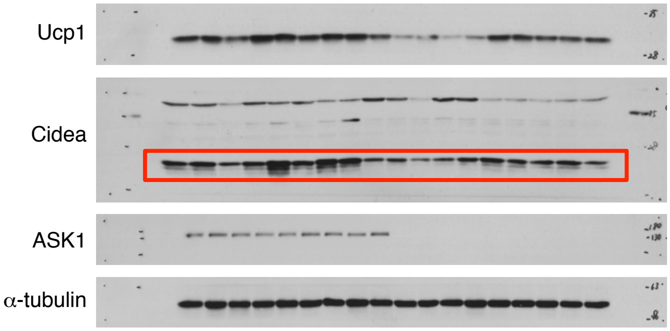

Fig. 2b

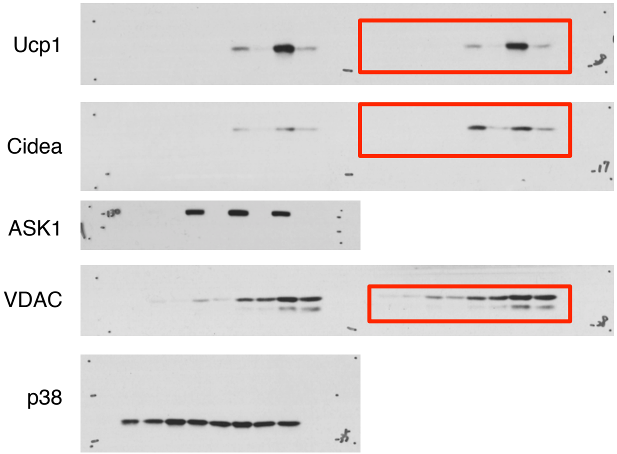

Fig. 2c

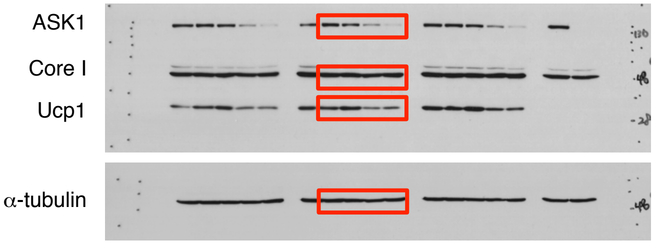

Fig. 2d

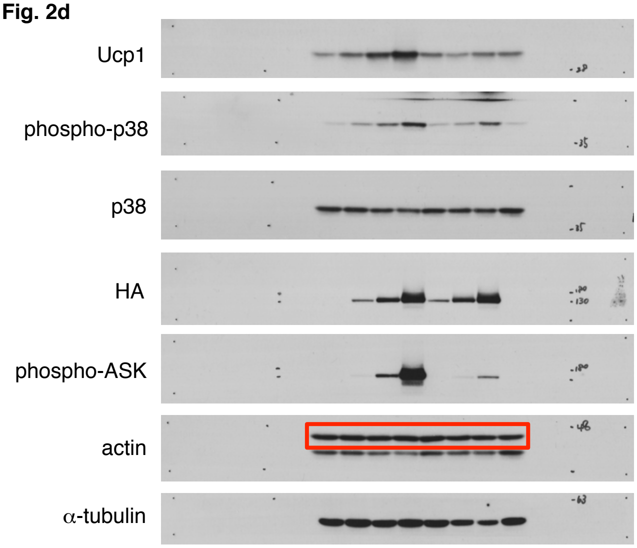

Fig. 3a

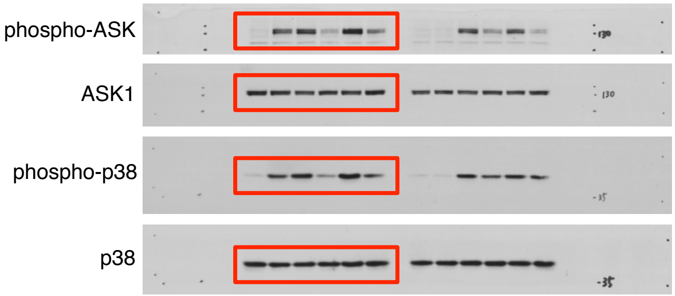

Supplementary Fig. 7 Uncropped scans of western blots displayed in the main figures

Fig. 3b

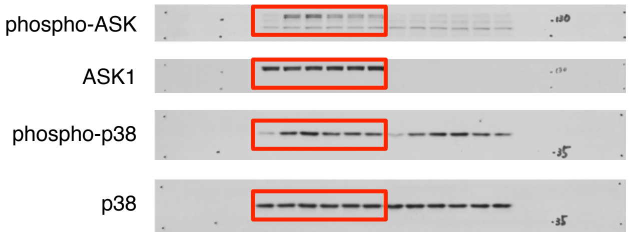

Fig. 3c, d

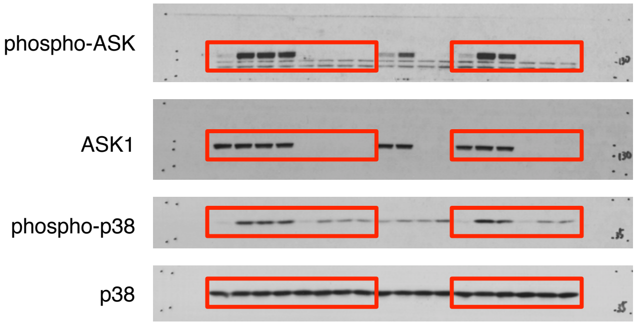

Fig. 3e

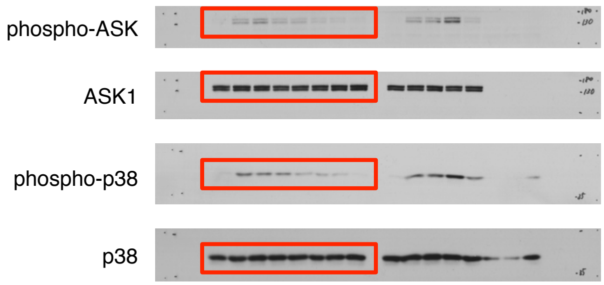

Fig. 3f

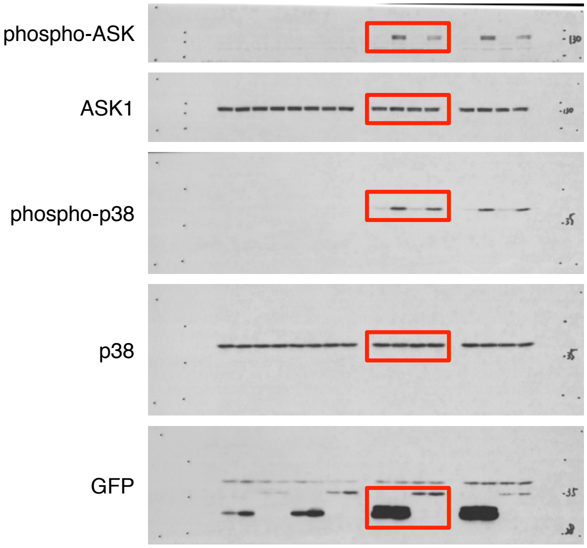

Fig. 3g

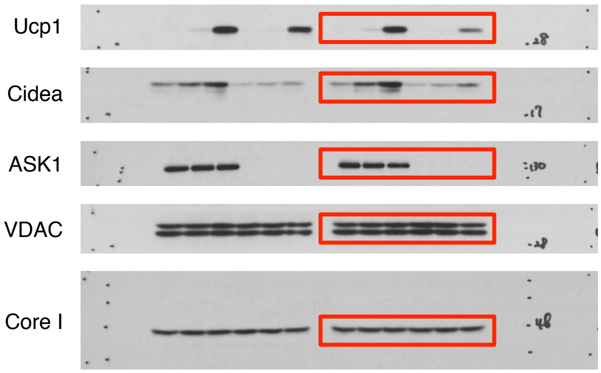

Fig. 4a

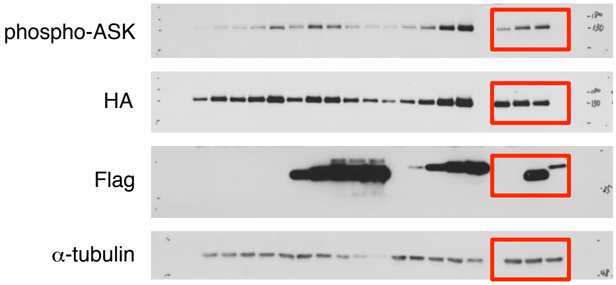

Fig. 4b

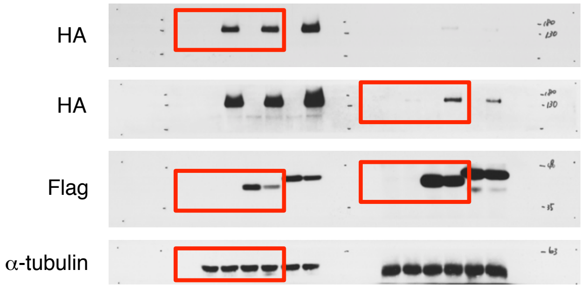

Supplementary Fig. 7 Uncropped scans of western blots displayed in the main figures

Fig. 4c

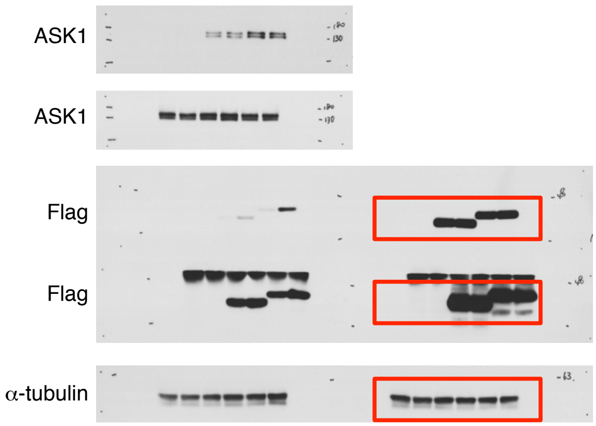

Fig. 4d

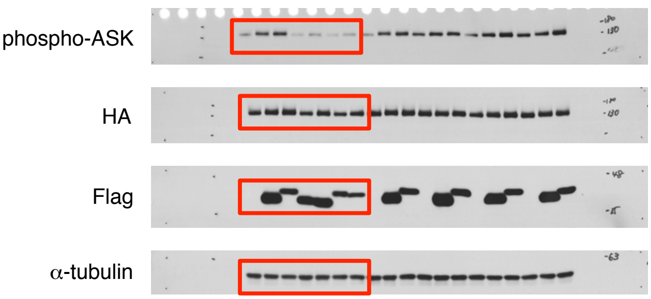

Fig. 5b

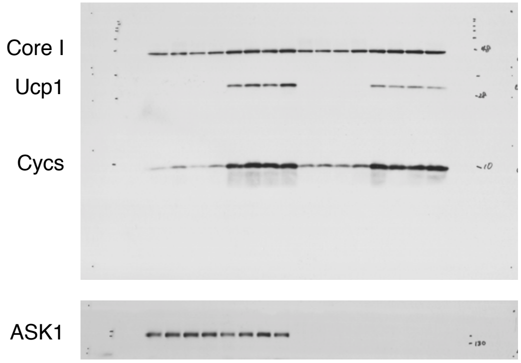

Fig. 5c

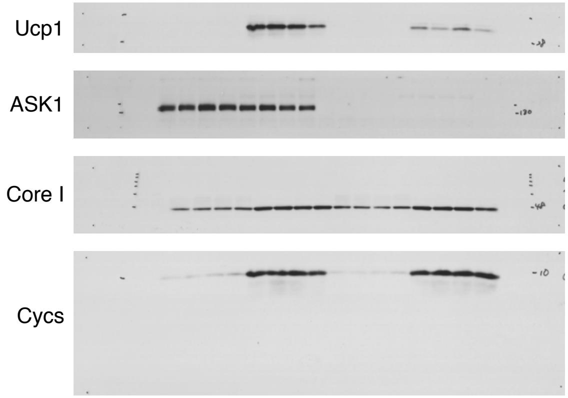

Fig. 5d

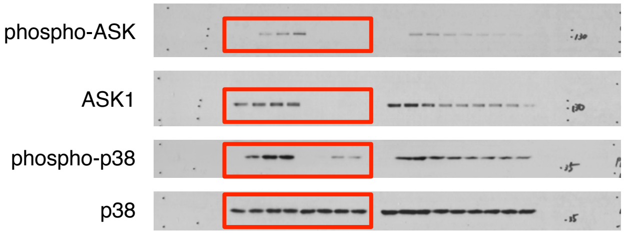

Fig. 5e

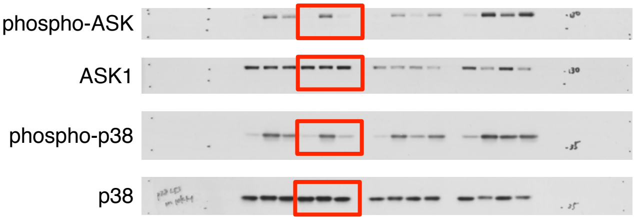

Fig. 5f

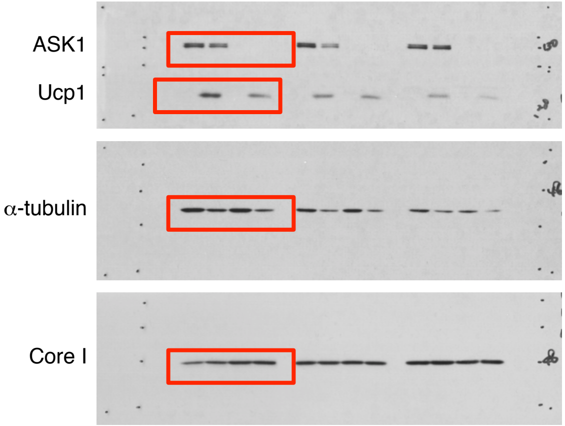

Supplementary Fig. 7 Uncropped scans of western blots displayed in the main figures

Supplementary Table 1 Gene expression ratios in ASK1-deficient iBAT identified by microarray analysis

gene	ratio
Ucp1	0.82372
Cidea	0.98971
Dio2	0.21137
Pgc1a	1.05517
Pgc1a	0.65673
Acox1	0.67968
Nr4a1	0.20565
Nr4a2	0.28227
Nr4a2	0.31835
Elovl3	0.05928

Supplementary Table 2 Gene expression ratios in ASK1-deficient eWAT identified by microarray analysis

gene	ratio
Ucp1	0.03956
Cidea	0.20874
Dio2	0.48402
Pgc1a	0.88707
Pgc1a	0.84705
Elovl3	0.13582
Tbx1	0.45635
CD40	0.63261

Supplementary Table 3 Primer Sequences

gene	Forward	Reverse
Ucp1	ggcctctacgactcagtcca	taagccggctgagatcttgt
Cidea	aaaccatgaccgaagtagcc	aggccagttgtgatgactaagac
Dio2	ctgcgctgtgtctggaac	ggagcatcttcacccagttt
Pgc1a	cagtcgcaacatgctcaag	tggggtcatttggtgactct
Prdm16	tctcggatcccatcctca	ggaagatcttgccacagtacct
Cox4i1	tcactgcgctcgttctgat	cgatcgaaagtatgagggatg
Cycs	aaatctccacggtctgttcg	ccaggtgatgcctttgttct
Cox8b	ccagccaaaactcccactt	gaaccatgaagccaacgac
Cox7a1	cgaagaggggaggtgactc	agcctgggagacccgtag
Nr4a1	ctgtccgctctggtcctc	aatgcgattctgcagctctt
Nr4a2	tcagagcccacgtcgatt	tagtcagggtttgcctggaa
Adipoq	ggagagaaaggagatgcaggt	ctttcctgccaggggttc
Plin1	ggatggagacctccctgag	ctcacaggtcccgctcac
Pparg	gaaagacaacggacaaatcacc	gggggtgatatgtttgaacttg
Ckm	cagcacagacagacactcagg	gaacttgttgtgggtgttgc
Elovl3	gaggcctctcatcctctggt	ttgccataaacttccacatcc
ASK1	cgtgctggaccgtttttac	tctcgcactccaagatggta
S18	tccagcacattttgcgagta	cagtgatggcgaaggctatt
